# Adaptive Natural Killer Cells Integrate Interleukin-18 during Target-Cell Encounter

**DOI:** 10.3389/fimmu.2017.01976

**Published:** 2018-01-17

**Authors:** Quirin Hammer, Timo Rückert, Josefine Dunst, Chiara Romagnani

**Affiliations:** ^1^Innate Immunity, German Rheumatism Research Center (DRFZ), Leibniz Association, Berlin, Germany; ^2^Inflammation Biology, German Rheumatism Research Center (DRFZ), Leibniz Association, Berlin, Germany; ^3^Medical Department I, Charité – University Medicine Berlin, Berlin, Germany

**Keywords:** human natural killer cells, adaptive natural killer cells, NKG2C, interleukin-18, costimulation

## Abstract

Human cytomegalovirus (HCMV) infection induces adaptations in the natural killer (NK)-cell compartment. Expanded subsets of adaptive NK cells display potent effector functions against cellular targets, despite their apparent unresponsiveness to stimulation with classical dendritic cell-derived cytokines interleukin (IL)-12 and IL-18. However, it remains unclear whether adaptive NK cells have completely lost their ability to sense inflammation *via* IL-12 and IL-18 or whether these pro-inflammatory signals can be functionally integrated into defined contexts. Here, we demonstrate that adaptive NKG2C^+^ NK cells can be costimulated by the presence of pro-inflammatory cytokines during target cell-induced activation. Cytokine costimulation of adaptive NK cells resulted in elevated interferon (IFN)-gamma and tumor necrosis factor (TNF) production, which promoted protein expression of HLA class I and adhesion molecules as well as transcription of genes involved in antigen processing and antiviral states in endothelial bystander cells *in vitro*. We further show that IL-18 drove costimulation in functional assays and was sufficient for elevated cytokine production in the absence of IL-12. Hence, adaptive NKG2C^+^ NK cells—although poorly responsive to IL-12 and IL-18 as an isolated stimulus—integrate IL-18 as a costimulatory signal during target-cell encounter.

## Introduction

Natural killer (NK) cells are lymphocytes required for proficient immunity against viral infections (1), and as members of the innate lymphoid cell family, NK cells are classically regarded part of the innate immune system (2). However, emerging experimental evidence suggest that NK cells can display adaptive-like features in response to inflammatory signals (3, 4), hapten challenge (5), and especially viral infection (6, 7).

In humans, adaptive NK-cell responses are driven by human cytomegalovirus (HCMV) infection and are associated with the expansion of subsets expressing the activating receptor NKG2C (8). Adaptive NKG2C^+^ NK cells in HCMV-seropositive individuals display skewed and narrow expression patterns of otherwise stochastically distributed killer Ig-like receptors, which is suggestive of oligoclonal or clonal-like expansion upon HCMV infection and parallels the pathogen-specific responses of T cells (9, 10). Furthermore, HCMV-induced adaptive NK cells display a remodeled epigenetic landscape analogous to the global differentiation program of effector-memory CD8^+^ T cells (11, 12). The loss of DNA methylation at regulatory regions within the *TNF* and *IFNG* loci is peculiarly shared between adaptive NK cells and terminally differentiated T cells (11, 12), enabling robust cytokine production and highlighting adaptive traits at the molecular level.

The functionality of adaptive NK cells is further calibrated by their activating receptor expression pattern, which determines their recognition properties [reviewed in Ref. (13)]. Adaptive NK cells largely lack natural cytotoxicity receptors such as NKp30 and NKp46, but preferentially express the activating receptor NKG2C and the costimulatory receptor CD2, while other activating receptors such as CD16 are similarly expressed by adaptive and conventional NK cells (8, 10, 14). Accordingly, adaptive NK cells proficiently produce cytokines upon engagement of NKG2C or CD16 by HLA-E-expressing or antibody-coated target cells, respectively (9), and cross-linking of CD2 can further amplify adaptive NK-cell functions (14). In contrast to conventional NK cells, adaptive NK cells were reported to display poor responsiveness toward the classical NK cell-activating dendritic cell-derived cytokines, interleukin (IL)-12 and IL-18 (9, 12), suggesting an altered recognition strategy poised for responses against defined cellular targets.

However, both infected cells and a robust inflammatory milieu are present during viral infection (15–17), and it remains incompletely understood whether adaptive NKG2C^+^ NK cells have completely lost their ability to sense IL-12 and IL-18 (IL-12 + 18) and rely solely on recognition of cellular stimuli, or whether adaptive NKG2C^+^ NK cells are able to functionally respond to these inflammatory cues in the context of target-cell encounter.

Here, we show that adaptive NKG2C^+^ NK cells are poorly responsive to IL-12 + 18 as a single stimulus, but if provided alongside target cells, IL-12 + 18 results in amplification of adaptive NKG2C^+^ NK-cell cytokine production. We further demonstrate that cytokine costimulated adaptive NKG2C^+^ NK cells relay enhanced activation to bystander cells and that IL-18 functionally drives elevated cytokine production during target-cell encounter.

## Results

### Effector Responses of Adaptive NK Cells against Target Cells Are Amplified by Cytokine Costimulation

**Figure 1:**
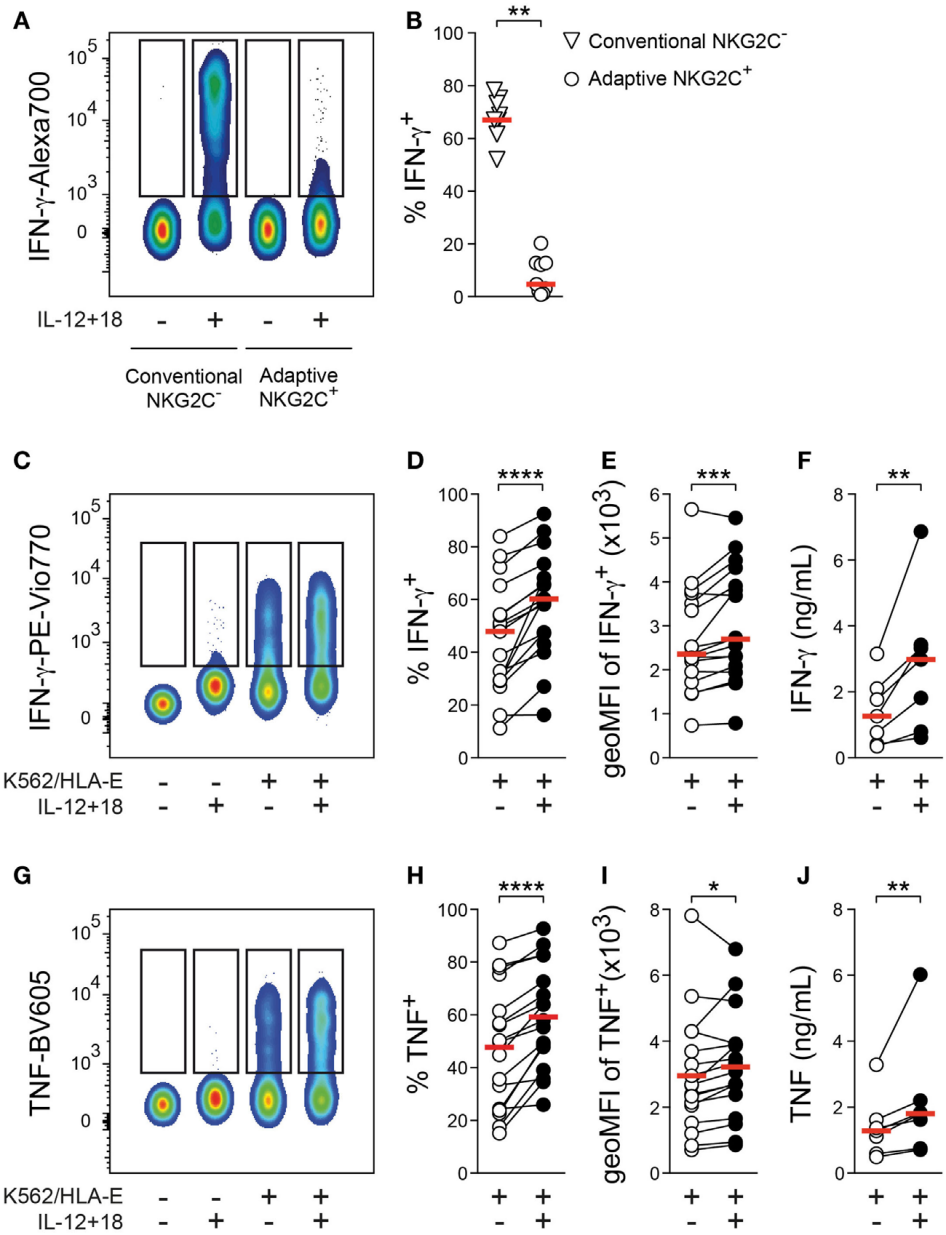
Effector responses of adaptive natural killer (NK) cells against target cells are amplified by cytokine costimulation. **(A)** Representative staining of interferon (IFN)-γ gated on conventional NKG2C^−^ or adaptive NKG2C^+^ NK cells after 24 h culture in the absence or presence of interleukin (IL)-12 + 18. **(B)** Summary of frequencies of IFN-γ^+^ cells. Symbols indicate individual donors, and red lines indicate median (*n* = 9). **(C)** Representative staining of IFN-γ gated on adaptive NKG2C^+^ NK cells after 6 h culture either alone or with K562/HLA-E target cells in the absence or presence of IL-12 + 18. **(D)** Summary of frequencies of IFN-γ^+^ cells and **(E)** summary of geometric mean fluorescence intensity (geoMFI) of IFN-γ in IFN-γ^+^ cells, *n* = 17 donors. **(F)** Summary of IFN-γ protein concentrations in the supernatant of cocultures, *n* = 7 donors. **(G)** Representative staining of tumor necrosis factor (TNF) gated on adaptive NKG2C^+^ NK cells after 6 h culture either alone or with K562/HLA-E target cells in the absence or presence of IL-12 + 18. **(H)** Summary of frequencies of TNF^+^ cells and **(I)** summary of geoMFI of TNF in TNF^+^ cells, *n* = 17 donors. **(J)** Summary of TNF protein concentrations in the supernatant of cocultures, *n* = 7 donors. Connected symbols indicate individual donors, and red lines indicate median. All statistical analyses performed with one-tailed Wilcoxon matched-pairs test. *p < 0.05, **p < 0.01, ***p < 0.001, and ****p < 0.0001.

Reprogrammed effector functions are a hallmark of adaptive NK cells and, in line with previous data (9, 12), only a minor fraction of adaptive NKG2C^+^ NK cells produced the NK-cell signature cytokine interferon (IFN)-γ after 24 h stimulation with IL-12 + 18 as compared to conventional NKG2C^−^ NK cells (Figures 1A,B), suggesting that adaptive NK cells are largely insensitive to these pro-inflammatory cytokines as a single stimulus.

To test whether adaptive NK cells have retained the capacity to integrate IL-12 + 18 as a costimulatory signal during target-cell encounter, we cultured FACS-purified NK cells with target cells in the absence or presence of IL-12 + 18. Adaptive NKG2C^+^ NK cells proficiently produced IFN-γ and tumor necrosis factor (TNF) upon stimulation with K562/HLA-E cells, which express HLA-E and engage the key activating receptor NKG2C (Figures 1C,G). Importantly, the presence of IL-12 + 18 during target-cell stimulation consistently increased the frequency of IFN-γ^+^ as well as TNF^+^ adaptive NK cells (Figures 1D,H), indicating that these pro-inflammatory cytokines can still function as costimulatory signals for adaptive NK cells. Moreover, the addition of IL-12 + 18 resulted in elevated signal intensity of both IFN-γ and TNF (Figures 1E,I), and presence of IL-12 + 18 during target-cell encounter was associated with significantly heightened IFN-γ and TNF protein concentrations in the supernatant of cocultures (Figures 1F,J), thus further corroborating a costimulatory effect.

Increased frequencies of IFN-γ^+^ and TNF^+^ adaptive NK cells were also detected after culture with beads coated with an agonistic anti-NKG2C antibody in the presence of IL-12 + 18 (Figures S1D,E in Supplementary Material), suggesting that sole ligation of NKG2C is sufficient to enable costimulatory effects of IL-12 + 18.

Furthermore, elevated IFN-γ and TNF production was similarly observed after culture with rituximab-coated 721.221 B-cell lymphoma cells in the presence of IL-12 + 18 (Figures S1F,H in Supplementary Material), implying that potentiation of cytokine production by pro-inflammatory signals is not restricted to NKG2C-mediated activation but also detectable upon engagement of other adaptive NK-cell activating receptors such as CD16.

Altogether, these data demonstrate that—although largely unresponsive to IL-12 + 18 as an isolated stimulus—adaptive NK cells can integrate IL-12 + 18 into effector responses against target cells, resulting in augmented cytokine production.

### Cytokine Costimulated Adaptive NK Cells Proficiently Alert Bystander Cells *In Vitro*

**Figure 2:**
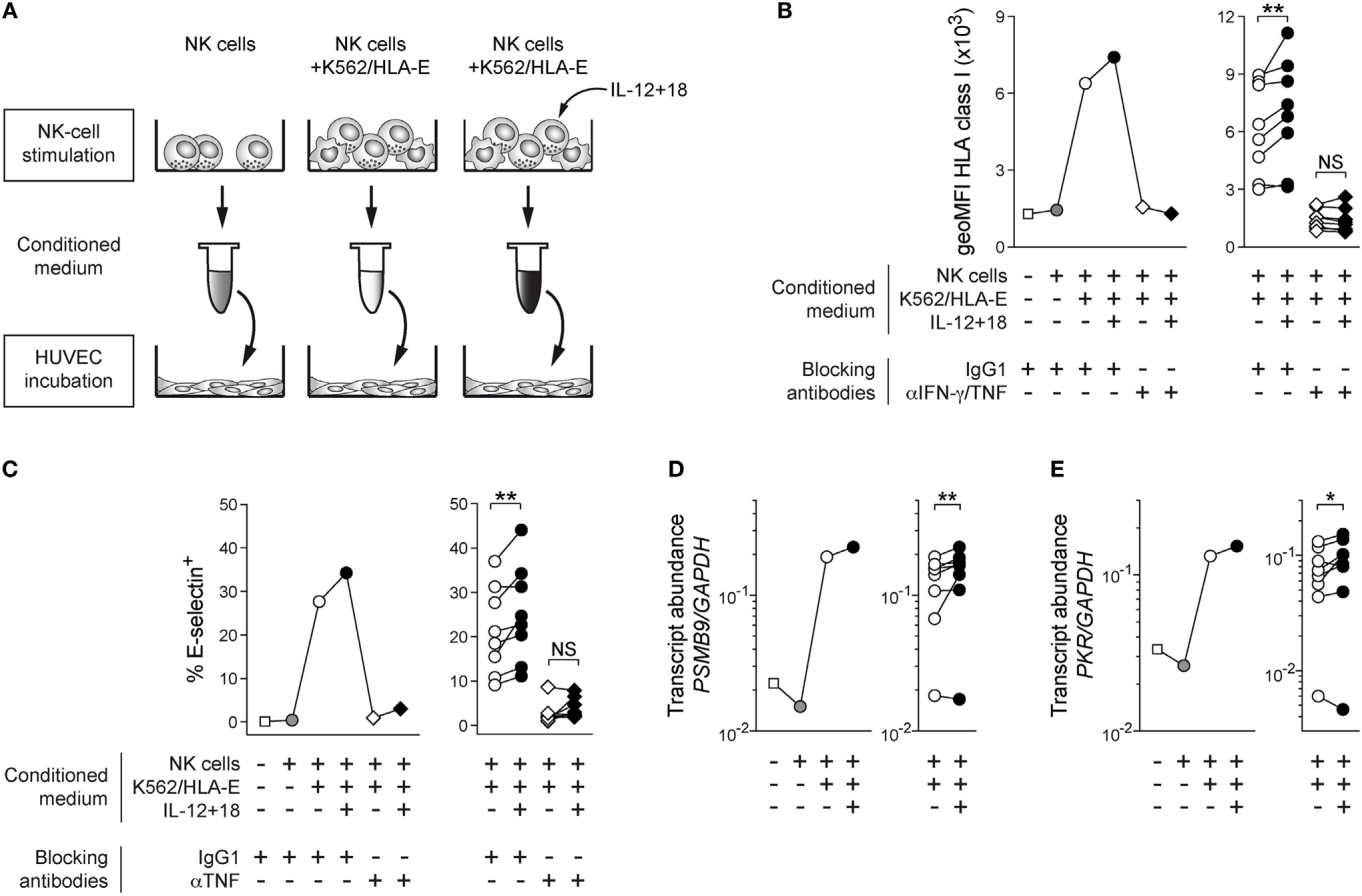
Cytokine costimulated adaptive natural killer (NK) cells proficiently alert bystander cells *in vitro*. **(A)** Schematic illustration of experimental design. **(B)** Representative geoMFI of HLA class I on human umbilical vein endothelial cells (HUVEC) after 40 h treatment with indicated conditioned medium in the presence or absence of anti-interferon (IFN)-γ and anti-tumor necrosis factor (TNF) blocking antibodies (left) and summary of *n* = 8 treatments with conditioned medium from independent NK-cell stimulations (right). **(C)** Representative frequency of E-selectin^+^ HUVEC after 6 h treatment with indicated conditioned medium in the presence or absence of anti-TNF blocking antibodies (left) and summary of *n* = 8 treatments with conditioned medium from independent NK-cell stimulations (right). **(D)** Representative *PSMB9* transcript abundance relative to *GAPDH* in HUVEC after 24 h treatment with indicated conditioned medium (left) and summary of *n* = 8 NK-cell stimulations (right). **(E)** Representative protein kinase R (PRK) *PKR* transcript abundance relative to *GAPDH* in HUVEC after 24 h treatment with indicated conditioned medium (left) and summary of *n* = 8 treatments with conditioned medium from independent NK-cell stimulations (right). Connected symbols indicate individual donors used to generate conditioned medium. All statistical analyses performed with one-tailed Wilcoxon matched-pairs test. NS, not significant; *p < 0.05 and **p < 0.01.

Natural killer cell-derived IFN-γ and TNF mediate pleiotropic effects on both immune and non-immune bystander cells such as endothelial cells, which can be alerted to participate in immune responses as conditional antigen-presenting cells due to their ability to present antigen and regulate influx of antigen-specific lymphocytes *via* adhesion molecules (18–21). To test the functional capacity of IL-12 + 18 costimulated adaptive NK cells and to investigate whether the integration of pro-inflammatory signals during target-cell recognition can be relayed to bystander cells, human umbilical vein endothelial cells (HUVEC) were treated with conditioned medium obtained from supernatants of FACS-sorted adaptive NKG2C^+^ NK cells cocultured with K562/HLA-E either in the absence or presence of IL-12 + 18 (Figure 2A). In line with the reported contribution of IFN-γ and TNF in activating endothelial cells (18, 21), medium conditioned by K562/HLA-E-stimulated adaptive NK cells induced clear upregulation of HLA class I protein on HUVEC (Figure 2B). Importantly, HUVEC responded to conditioned medium from IL-12 + 18 costimulated adaptive NK cells with consistently higher HLA class I expression (Figure 2B) while addition of IL-12 + 18 directly to HUVEC had no effect (Figure S2A in Supplementary Material), suggesting that increased cytokine output resulting from IL-12 + 18 costimulation of adaptive NKG2C^+^ NK cells can be relayed to bystander cells.

Increase of HLA class I protein levels was blocked upon addition of anti-IFN-γ and anti-TNF antibodies to HUVEC, indicating that NK cell-secreted IFN-γ and TNF played major roles in mediating endothelial-cell activation (Figure 2B; Figure S2B in Supplementary Material).

Furthermore, TNF-dependent induction of E-selectin surface expression was consistently elevated after incubation with conditioned medium from adaptive NK cells costimulated by IL-12 + 18 (Figure 2C; Figure S2C in Supplementary Material). HUVEC incubated with conditioned medium of stimulated adaptive NK cells homogenously expressed ICAM-1 at the cell surface, and its expression levels were mildly increased when adaptive NK cells were costimulated with exogenous cytokines (Figure S2D in Supplementary Material), while VCAM-1 was not preferentially affected (Figure S2E in Supplementary Material).

In agreement with endothelial-cell activation, transcripts of *B2M* (encoding the β_2_ microglobulin component of HLA class I heterodimers) and *PSMB9* (encoding the inducible immunoproteasome subunit β9) were induced in HUVEC treated with medium conditioned by K562/HLA-E-activated adaptive NKG2C^+^ NK cells and further upregulated when IL-12 + 18 was present during NK-cell stimulation (Figure 2D; Figure S2F in Supplementary Material), indicating that transcription of genes involved in both antigen presentation and antigen processing was preferentially triggered in HUVEC when adaptive NK cells integrated inflammatory signals during target-cell encounter.

Additionally, we observed that expression of the inducible antiviral effector molecule protein kinase R [PKR; (18)] was similarly elevated at the mRNA level (Figure 2E), implying that IL-12 + 18-mediated costimulation of adaptive NKG2C^+^ NK cells can efficiently prime bystander cells to acquire antiviral states.

Thus, these data suggest that the presence of IL-12 + 18 during target-cell encounter has functional consequences and enables adaptive NKG2C^+^ NK cells to proficiently alert bystander endothelial cells by instructing enhanced protein expression of HLA class I and adhesion molecules as well as promote transcription of genes involved in antigen processing and antiviral functions.

### IL-18 Drives Costimulation of Adaptive NK Cells

**Figure 3:**
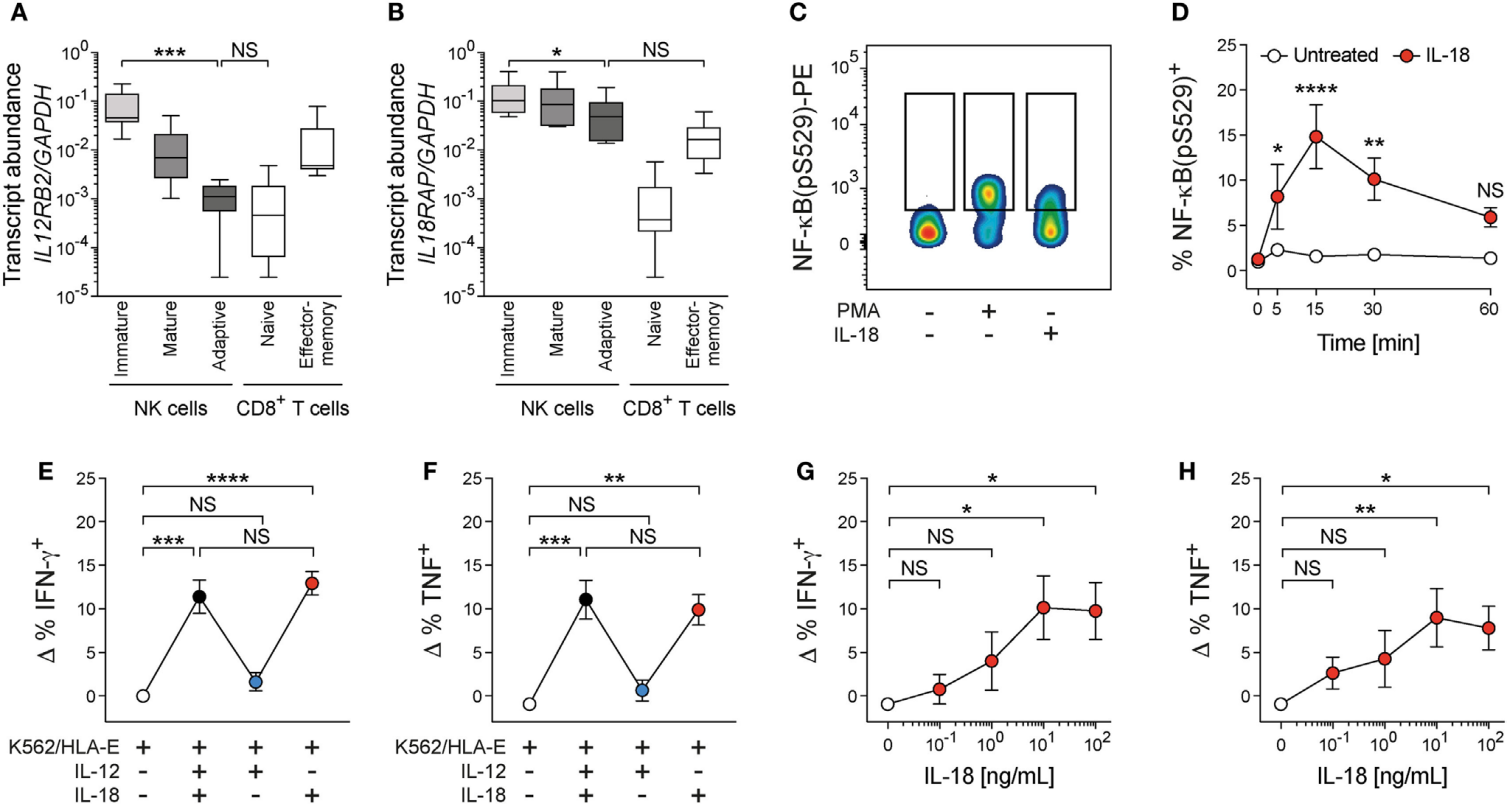
Interleukin (IL)-18 drives costimulation of adaptive natural killer (NK) cells. **(A)** *IL12RB2* and **(B)** *IL18RAP* transcript abundance relative to *GAPDH* in *ex vivo* FACS-purified immature conventional NK cells (CD56^dim^ CD57^−^ NKG2C^−^), mature conventional NK cells (CD56^dim^ CD57^+^ NKG2C^−^), adaptive NKG2C^+^ NK cells (CD56^dim^ CD57^+^ NKG2C^+^), naïve T cells (CD3^+^ CD8^+^ CD45RA^+^ CD45RO^−^ CCR7^+^), and effector-memory T cells (CD3^+^ CD8^+^ CD45RA^−^ CD45RO^+^ CCR7^−^), *n* = 6 individual donors. Box plots display minimum to maximum and median. Statistical analysis performed with Friedman and Dunn’s multiple comparison test. **(C)** Representative staining of NF-κB(pS529) in FACS-purified adaptive NKG2C^+^ NK cells after treatment with medium, phorbol-12-myristat-13-acetat (PMA), or interleukin (IL)-18 for 15 min. **(D)** Summary of frequencies of NF-κB(pS529)^+^ cells either treated with medium or IL-18 over time, *n* = 5 donors. Symbols indicate mean and error bars SEM. Statistical analysis performed with repeated-measures two-way ANOVA with Bonferroni correction. **(E,F)** NK cells were cultured with K562/HLA-E in the presence of IL-12 + 18, IL-12, or IL-18. **(E)** Frequencies of ΔIFN-γ^+^ [calculated by subtraction of the frequency of interferon (IFN)-γ^+^ cells in the presence of K562/HLA-E alone] and **(F)** ΔTNF^+^ adaptive NK cells, *n* = 12 donors. Symbols indicate mean and error bars SEM. Statistical analysis performed with Friedman and Dunn’s multiple comparison test. **(G,H)** NK cells were cultured with K562/HLA-E and varying concentrations of IL-18. **(G)** Frequencies of ΔIFN-γ^+^ and **(F)** ΔTNF^+^ adaptive NK cells, *n* = 6 donors. Symbols indicate mean and error bars SEM. Statistical analysis performed with Friedman and Dunn’s multiple comparison test. NS, not significant; *p < 0.05, **p < 0.01, ***p < 0.001, and ****p < 0.0001.

Since our data point toward a merely costimulatory function of IL-12 + 18 for adaptive NK cells, we sought to better understand the underlying regulation. We therefore analyzed the transcript expression of *IL12RB2* and *IL18RAP* in *ex vivo* FACS-sorted terminally differentiated adaptive NKG2C^+^ NK cells (CD56^dim^ CD57^+^ NKG2C^+^), mature conventional NK cells (CD56^dim^ CD57^+^ NKG2C^−^), and immature conventional NK cells (CD56^dim^ CD57^−^ NKG2C^−^) together with naïve (CD45RA^+^ CD45RO^−^ CCR7^+^) and effector-memory (CD45RA^−^ CD45RO^+^ CCR7^−^) CD8^+^ T cells (Figures S3A,B in Supplementary Material). In this setting, naïve CD8^+^ T cells serve as a negative control due to their unresponsiveness to IL-12 + 18, while effector-memory CD8^+^ T cells profoundly produce IFN-γ upon stimulation with IL-12 + 18 *in vitro* and *in vivo* (22–24). In line with previous data (12), *IL12RB2* transcripts were clearly reduced in adaptive NKG2C^+^ NK cells compared to immature NK cells (41-fold decrease in median transcript abundance), approaching the expression level of naïve CD8^+^ T cells (Figure 3A). *IL18RAP* mRNA was also reduced in adaptive NKG2C^+^ when compared to immature NK cells (Figure 3B); however, this reduction was modest (2.2-fold decrease in median transcript abundance) and *IL18RAP* transcript levels of adaptive NKG2C^+^ NK cells were not significantly different from those of effector-memory CD8^+^ T cells (Figure 3B). Furthermore, diminished *IL12RB2* transcript levels correlated with those of *ZBTB16* (encoding PLZF), while this was not the case for *IL18RAP* (Figure S3C in Supplementary Material), suggesting an altered cytokine receptor profile of adaptive NK cells, which might result in a selective unresponsiveness toward IL-12 and potentially involves distinct regulatory programs controlling the disparate expression of cytokine receptors.

In agreement with previous reports, IL-12 failed to transmit signals *via* phosphorylated STAT4, while treatment with IL-18 resulted in consistent phosphorylation of NF-κB in FACS-sorted adaptive NK cells [(12); Figures 3C,D; Figures S3D–G in Supplementary Material], implying that IL-18 could contribute to coactivate adaptive NK-cell effector responses.

To ascertain whether IL-18 is the decisive signal for amplifying cytokine responses, we dissected the individual contributions of IL-12 and IL-18 during target-cell encounter. Indeed, IL-18 drove costimulation of adaptive NK cells in functional assays and was sufficient to augment IFN-γ and TNF production during coculture with K562/HLA-E target cells or αNKG2C beads, while IL-12 did not impact on the frequencies of cytokine-producing cells (Figures 3E,F; Figures S3G,H in Supplementary Material). Functional responses of adaptive NK cells to IL-18 costimulation reached a plateau at 10 ng/mL (Figures 3G,H), suggesting that low doses of IL-18 can potentiate adaptive NK-cell activation. Conversely, addition of IL-12 to functional assays did not impact on adaptive NK-cell cytokine production, nor did IL-12 synergize with IL-18 for costimulation (Figures 3E,F; Figures S3G,H in Supplementary Material).

Collectively, these data demonstrate that the presence of pro-inflammatory cytokines during target-cell encounter results in amplified IFN-γ and TNF production by adaptive NK cells. The amplified activation can be relayed to bystander endothelial cells, which in turn acquire elevated antigen processing and presentation capacity as well as antiviral states. Selective responsiveness toward IL-18 but not IL-12 for costimulation of target cell-induced activation highlights the refined receptor expression pattern of adaptive NK cells and further emphasizes their restricted recognition strategy, which enables adaptive NK cells to proficiently respond to finely selected stimuli.

## Discussion

Adaptive NKG2C^+^ NK cells are potent effectors against HLA-E-expressing and antibody-coated target cells while considered insensitive to innate cues such as the pro-inflammatory cytokines IL-12 and IL-18. Yet, it is not known whether adaptive NK cells have completely lost the responsiveness to IL-12 and IL-18 or whether they can respond to inflammatory signals in a specific context. Here, we demonstrate that IL-18 can potentiate adaptive NKG2C^+^ NK-cell activation elicited by target cells. The presence of IL-18 during target-cell encounter augments adaptive NK-cell cytokine production, likely by phosphorylation of NF-κB, which potentially synergizes with NKG2C- or CD16-mediated signaling for optimal IFN-γ and TNF release.

Interestingly, murine adaptive Ly49H^+^ NK cells arising in response to MCMV infection (6) are strictly dependent on IL-12 for their generation (25). However, when fully differentiated, murine adaptive Ly49H^+^ NK cells respond poorly to cytokine-mediated activation caused by Listeria or influenza infection *in vivo*, and their responsiveness to IL-12 *in vitro* is drastically diminished, although not completely abolished (26). Indeed, residual STAT4 phosphorylation is detectable and cross-linking of Ly49H can be costimulated by IL-12 *in vitro* (26). In contrast, likely due to drastic downregulation of *IL12RB2* transcripts, human adaptive NKG2C^+^ NK cells have fully lost their ability to respond to IL-12: IL-12 does not induce phosphorylation of STAT4 nor promote elevated IFN-γ or TNF production during target-cell encounter. Conversely, *IL18RAP* transcript levels are sustained in adaptive NKG2C^+^ NK cells and, although phosphorylation of NF-κB is less efficient in adaptive compared to conventional NK cells, IL-18 is preserved as a functional costimulatory signal for adaptive NK-cell effector responses. The disparate expression levels of *IL12RB2* and *IL18RAP* parallel and extend the previously described skewed receptor pattern of adaptive NK cells [reviewed in Ref. (13)]: adaptive NK cells express reduced frequencies of activating receptors such as NKp30 or NKp46 compared to conventional NK cells, while maintaining CD16 and preferentially expressing NKG2C, which restrains the recognition properties of adaptive NK cells. Consequently, the restricted recognition repertoire renders adaptive NK cells especially responsive to specific stimuli such as HLA-E-expressing or antibody-coated targets, while cells expressing ligands for NKp30 or NKp46 inadequately trigger adaptive NK-cell functions. Similarly, IL-18 costimulation results in amplified cytokine production, whereas IL-12 has no functional impact.

Selective sensing of IL-18 but not IL-12 by human adaptive NK cells is of particular interest since multiple infections and inflammatory diseases are associated with systemic or local cytokine induction (27, 28). In this context, unresponsiveness of HCMV-induced adaptive NK cells to IL-12 might guard the population against IL-12-mediated activation-induced cell death during unrelated infections (29), thereby contributing to long-term maintenance of adaptive NK cells (30, 31). Intriguingly, IL-18 is a central component of the global cytokine signature detected in patients experiencing HCMV reactivation (32), which suggests that HCMV-infected host cells and systemic IL-18 are concomitantly present during acute infection. The presence of varying IL-18 levels during target-cell encounter potentially fine-tunes cytokine secretion of adaptive NKG2C^+^ NK cells and enables optimal effector functions. While previous studies have demonstrated that CD56^dim^ but not CD56^bright^ NK cells benefit from addition of IL-12 + 18 during target-cell encounter (33), selective costimulation *via* IL-18 but not IL-12 is exclusive to adaptive NK cells and implies a pivotal role of IL-18 in modulating the functional response of this NK-cell subset. Notably, IL-18 was reported to synergize with low doses of common γ chain cytokines IL-2, IL-15, and IL-21 in potentiating NK-cell activation (34), and the description of IL-1R8 as a negative regulator of the IL-18-IL-18R axis (35) highlights the prominent contribution of IL-18 to antiviral as well as antitumor NK-cell effector functions. Thus, IL-18 might act as a key indicator of inflammatory burden for adaptive NK cells and calibrate their cytokine production accordingly.

NKG2C expression marks the majority of adaptive NK-cell populations, but HCMV-induced NKG2C^−^ adaptive NK cells have been described in *KLRC2* (encoding NKG2C)-sufficient and -deficient humans (8, 10, 14, 36). In agreement with these studies, NKG2C cross-linking was sufficient but not required to enable costimulation by exogenous cytokines, since CD16-dependent activation of adaptive NK cells by rituximab-coated 721.221 target cells similarly benefited from costimulation. Based on these data, we expect that other activating receptors, which can efficiently trigger IFN-γ and TNF production of NKG2C^−^ adaptive NK cells, could be functionally calibrated by concomitant IL-18 signaling.

Although both EBV (37) and chronic hepatitis (38) were reported to not affect adaptive NK-cell expansions in HCMV-seropositive individuals, provision of ample costimulatory signals—by cytokines and/or cellular ligands—could permit the activation of pre-existing HCMV-induced adaptive NK cells during heterologous infections including Hantavirus (39), HIV-1 (8, 40), or Chikungunya-virus infection (41).

In summary, our study revealed that adaptive NKG2C^+^ NK cells can indeed be costimulated by IL-18 during target-cell encounter, resulting in augmented cytokine production. We propose that adaptive NK cells may act as a target cell-restricted inflammation-sensing immune hub, which is able to relay pro-inflammatory signals to bystander cells and thereby potentially amplify ensuing immune responses.

## Materials and Methods

### Human Samples

Buffy coats of HCMV-seropositive healthy blood donors were obtained from the German Red Cross as approved by Charité ethics commission (EA1/149/12). PBMCs were isolated by density gradient centrifugation (Ficoll Paque Plus, GE Healthcare) and screened for the presence of adaptive NKG2C^+^ NK cells as previously described (42). In brief, coexpression analysis was employed to detect adaptive CD2^+^ CD57^+^ ILT2^+^ Siglec-7^−^ NKp30^−^ NKG2A^−^ within the CD56^dim^ NKG2C^+^ population (Figures S1A,B in Supplementary Material). Subsequently, CD56^+^ cells were MACS-enriched (CD56 MicroBeads, Miltenyi Biotec) from donors containing adaptive NK-cell populations and cryopreserved in fetal bovine serum (FBS; Biowest) containing 10% DMSO (Sigma). In the following assays, adaptive NK cells were gated as CD56^dim^ CD57^+^ NKG2A^−^ NKG2C^+^ cells (Figure S1C in Supplementary Material).

### Cells, Cell Lines, and HLA-E Surface Stabilization

Primary HUVEC (Lonza) were maintained in endothelial growth medium (Lonza or PromoCell) containing Vascular Endothelial Growth Factor according to the manufacturer’s instructions. 721.221 were maintained in complete medium [RPMI-1640 containing glutamine and supplemented with 10% (v/v) FBS, 20 µM β-mercaptoethanol, and 100 U/mL penicillin–streptomycin; all Thermo Fisher] and K562 transfected with HLA-E*01:03 [K562/HLA-E; provided by E. Weiss (43)] were maintained in complete medium containing 1 mg/mL Geneticin (InVivogen). Prior to functional assays, surface expression of HLA-E by K562/HLA-E was stabilized by pulsing with 300 µM synthetic HLA-Cw*01 signal peptide VMAPRTLIL (Peptides&Elephants) for 16–18 h in serum-free Opti-MEM (Thermo Fisher), similar to previous reports (44).

### Flow Cytometry

Single cell suspensions were stained with different combinations of fluorochrome-conjugated antibodies (Table S1 in Supplementary Material), and dead cells were excluded using Fixable Dead Cell Stain Kit (Thermo Fisher) or Fixable Viability Kit (BioLegend). For intracellular staining of IFN-γ and TNF, cells were surface stained and fixated with 2% PFA (EMS Sciences), followed by treatment with Permeabilizing Solution 2 (BD Biosciences). Data were acquired on an LSR Fortessa (BD Biosciences) and analyzed with FlowJo vX (FlowJo LLC) software. ARIA or ARIA II instruments (both BD Biosciences) were used for cell sorting experiments.

### Cytokine Stimulations

To assess IFN-γ production upon stimulation with cytokines, CD56^+^ MACS-enriched cells were thawed, FACS-purified for viable CD3^−^ CD56^+^ NK cells, and cultured in complete medium in the presence or absence of 10 ng/mL human recombinant IL-12 (Miltenyi Biotec) and 100 ng/mL IL-18 (MBL) for 24 h. GolgiPlug and GoligStop (both BD Biosciences) were added according to the manufacturer’s instructions and present during the last 5 h of the stimulation.

### Functional Assays and Cytokine Costimulation

For functional assays, CD56^+^ MACS-enriched cells were thawed, FACS-purified for viable CD3^−^ CD56^+^ NK cells, and rested overnight in complete medium. The next day, 2 × 10e5 NK cells were mixed with 1 × 10e5 target cells in V-bottom 96-well plates and the indicated concentrations of IL-12 and/or IL-18 were added. For cross-linking of CD16, rituximab (Roche) was present during the assay at the indicated concentrations. For sole engagement of NKG2C, MACSi beads (Miltenyi) were coated with an agonistic anti-NKG2C antibody (R&D Systems; biotinylated *in-house*) as previously described (11), and 1 × 10e6 αNKG2C beads were added to 2 × 10e5 NK cells. After 1 h incubation, GolgiPlug and GolgiStop (both BD Biosciences) were added according to the manufacturer’s instructions, and the assay continued for 5 h.

### Protein Measurements and Generation of Conditioned Medium

To measure protein concentrations in supernatants, CD56^+^ MACS-enriched cells were thawed, FACS-purified for viable CD3^−^ CD56^dim^ CD57^+^ NKG2C^+^ adaptive NK cells, and rested overnight in complete medium. The next day, 2 × 10e5 adaptive NK cells were mixed with 1 × 10e5 K562/HLA-E target cells in V-bottom 96-well plates in the absence or presence of IL-12 + 18. After 6 h of culture, cell-free supernatant was obtained by repeated centrifugation and stored at −80°C. IFN-γ and TNF protein levels were measured using LegendPlex (BioLegend) according to the manufacturer’s instructions.

Conditioned medium was obtained by mixing thawed supernatants 1:4 with EGM-2 for direct use in bystander cell assays.

### Bystander Cell Assays

For bystander cell assays, 2.5 × 10e4 HUVEC were allowed to adhere in 48-well plates in EGM-2, followed by treatment with conditioned medium in the presence of 10 µg/mL blocking anti-IFN-γ (clone B27) and anti-TNF (clone Mab1) or IgG1 isotype control (clone MPOC-21; all BioLegend) antibodies. When indicated, HUVEC were directly treated with 10 ng/mL IL-12 and 100 ng/mL IL-18, 1 ng/mL IFN-γ (R&D Systems), or 1 ng/mL TNF (kindly provided by AA Kruglov). To determine HLA class I expression, HUVEC were recovered from plates by trypsinization at 37°C after 40 h of treatment with conditioned medium, followed by FACS staining. Analysis of adhesion molecule expression by FACS was performed after 6 h incubation with conditioned medium and recovery of HUVEC using Accutase (Sigma) at room temperature. For assessing transcript levels, HUVEC were lysed after 24 h of treatment, and total RNA was isolated using RNeasy Micro Plus Kit (Qiagen) according to the manufacturer’s instructions.

### *Ex Vivo* Sorting of NK- and T-Cell Subsets

For *ex vivo* sorting experiments, NK and T cells were enriched from freshly isolated PBMC using CD56 Microbeads and CD8 Microbeads (both Miltenyi), respectively. MACS-enriched cells were sorted to >95% purity as immature conventional (CD3^−^ CD56^dim^ CD57^−^ NKG2C^−^) NK cells, mature conventional (CD3^−^ CD56^dim^ CD57^+^ NKG2C^−^) NK cells, adaptive (CD3^−^ CD56^dim^ CD57^+^ NKG2C^+^) NK cells, naïve (CD3^+^ CD4^−^ CD8^+^ CD45RA^+^ CD45RO^−^ CCR7^+^) CD8^+^ T cells, and effector-memory (CD3^+^ CD4^−^ CD8^+^ CD45RA^−^ CD45RO^+^ CCR7^−^) CD8^+^ T cells. Total RNA was isolated using the AllPrep DNA/RNA Mini Kit (Qiagen) according to the manufacturer’s instructions.

### Quantitative RT-PCR

To determine transcript levels, 50 ng of total RNA was reverse transcribed using TaqMan Reverse Transcription Reagents (Applied Biosciences), and quantitative real-time PCR was performed with TaqMan gene expression assays (Table S2 in Supplementary Material) on a StepOnePlus instrument (Applied Biosciences).

### Signaling Assays

To investigate phosphorylation of NF-κB and STAT4, CD56^+^ MACS-enriched cells were thawed, FACS-purified for viable CD3^−^ CD56^dim^ CD57^+^ NKG2C^+^ adaptive NK cells, CD56^dim^ CD57^−^ immature NK cells, or CD56^dim^ CD57^+^ mature NK cells, and rested overnight in complete medium. The next day, 0.25–1 × 10e5 NK cells were either treated with complete medium, 50 ng/mL phorbol-12-myristat-13-acetat, 100 ng/mL IL-18, or 100 ng/mL IL-12 for the indicated time periods and directly fixated in Cytofix (BD Biosciences). Fixated cells were permeabilized in PermIII (BD Biosciences) on ice, followed by staining of NF-κB (pS529) or STAT4 (pY693) (Table S1 in Supplementary Material).

### Statistical Analysis

Data were analyzed using GraphPad Prism 7. Wilcoxon matched-pairs test was applied to compare two groups of paired samples, while Friedman and Dunn’s multiple comparison test was used to compare three or more paired groups. When evaluating paired data sets with two variables (e.g., treatment and time), repeated-measures two-way ANOVA with Bonferroni correction was employed to determine statistical significance at each time point. NS indicates not significant, *p < 0.05, **p < 0.01, ***p < 0.001, and ****p < 0.0001.

## Ethics Statement

Buffy coats of HCMV-seropositive healthy blood donors were obtained from the German Red Cross as approved by Charité ethics commission (EA1/149/12).

## Author Contributions

QH conceived, designed, and administrated the study. QH, TR, and JD performed experiments and analyzed data. QH and CR interpreted data and drafted the manuscript. CR supervised the work. All the authors read and approved the submitted version of the manuscript.

## Conflict of Interest Statement

The authors declare that the research was conducted in the absence of any commercial or financial relationships that could be construed as a potential conflict of interest.
