# Supplementary material for: Adaptive Natural Killer Cells Integrate Interleukin-18 during Target-Cell Encounter
Source: Front Immunol. 2018 Jan 17;8:1976. doi: 10.3389/fimmu.2017.01976 (PMC5776097; doi:10.3389/fimmu.2017.01976)
Supplement: Supplementary file 1 [file Presentation_1.PDF]

Supplementary Material

Adaptive natural killer cells integrate interleukin-18 during target-cell encounter

Quirin Hammer*, Timo Rückert, Josefine Dunst, and Chiara Romagnani

*** Correspondence:** Quirin Hammer (quirin.hammer@drfz.de)

1 Supplementary Figures and Tables

1.1 Supplementary Figures

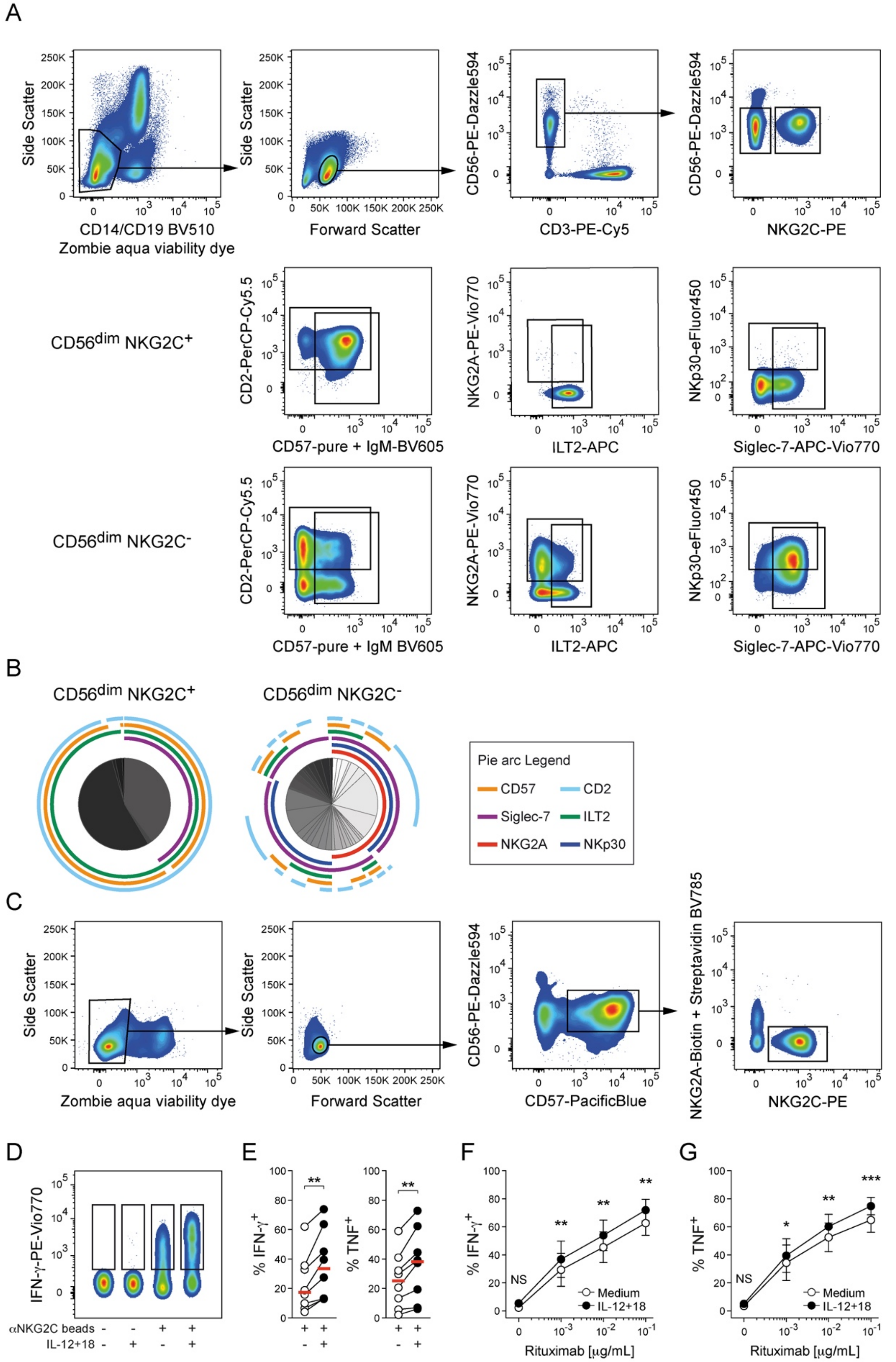

Supplementary Figure 1. Selection of donors with adaptive NKG2C^{+} NK cells and IL-12+18 co-stimulation of anti-NKG2C beads as well as antibody-coated target cells. (A-B) PBMC of HCMV-seropositive healthy donors were screened for adaptive NKG2C^{+} NK-cell populations. **(A)**

Representative gating to assess the expression of CD57, CD2, ILT2, NKG2A, Siglec-7, and NKp30 by $CD56^{dim}$ $NKG2C^{+}$ and $CD56^{dim}$ $NKG2C^{-}$ NK cells. **(B)** SPICE plots display co-expression pattern and enable detection of $CD2^{+}$ $CD57^{+}$ $ILT2^{+}$ Siglec-7^{-} $NKp30^{-}$ $NKG2A^{-}$ adaptive NK cells within the $CD56^{dim}$ $NKG2C^{+}$ population. **(C)** Representative gating of adaptive $CD56^{dim}$ $CD57^{+}$ $NKG2A^{-}$ $NKG2C^{+}$ NK cells in functional assays. (**D-E**) NK cells were incubated with beads coated with agonistic anti-NKG2C antibody in the absence or presence of IL-12+18. (**D**) Representative staining of IFN-γ gated on $CD56^{dim}$ $CD57^{+}$ $NKG2A^{-}$ KIR^{+} NK cells to enrich for adaptive $NKG2C^{+}$ NK cells. (**E**) Summary of frequencies of IFN-γ^{+} (left) and TNF^{+} (right) cells; n=8 donors. Connected symbols indicate individual donors and red lines median. Statistical analyses performed with one-tailed Wilcoxon matched-pairs test. **(F-G)** NK cells were co-cultured with 721.221 target cells in the absence or presence of IL-12+18 and varying concentrations of Rituximab. **(F)** Summary of frequencies of IFN-γ^{+} and **(G)** TNF^{+} adaptive $NKG2C^{+}$ NK cells; n=6 donors. Symbols indicate mean and error bars SEM. Statistical analysis performed with repeated-measures two-way ANOVA with Bonferroni correction. NS not significant, *$p < 0.05$, **$p < 0.01$, ***$p < 0.001$.

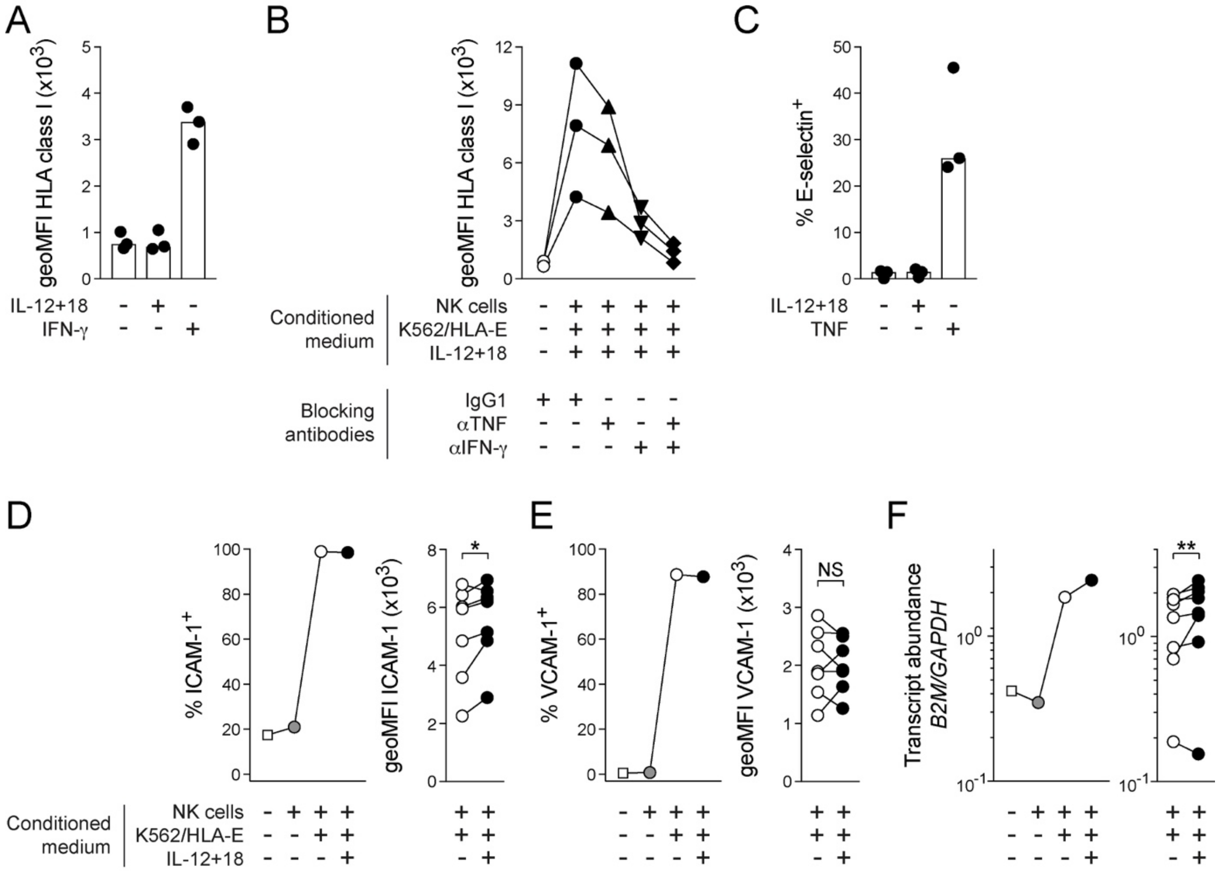

Supplementary Figure 2. IFN-γ and TNF but not IL-12+18 induce activation of HUVEC. (A) HLA class I geoMFI on HUVEC after treatment with IL-12+18 or IFN-γ for 40 h; n=3 independent experiments. Bars indicate median. (**B**) HLA class I geoMFI on HUVEC after treatment with conditioned medium in the absence or presence of anti-IFN-γ or anti-TNF blocking antibodies; n=3 treatments with conditioned medium from independent NK-cell stimulations. (**C**) Frequency of E-selectin$^+$ HUVEC after treatment with IL-12+18 or TNF for 6 h; n=3 independent experiments. Bars indicate median. (**D-E**) HUVEC were treated with indicated conditioned medium for 6 h followed by analysis of adhesion molecules. (**D**) Representative frequency of ICAM-1$^+$ HUVEC (left) and summary of ICAM-1 geoMFI on HUVEC of n=7 treatments with conditioned medium from independent NK-cell stimulations (right). (**E**) Representative frequency of VCAM-1$^+$ HUVEC (left) and summary of VCAM-1 geoMFI on HUVEC of n=7 treatments with conditioned medium from independent NK-cell stimulations (right). (**F**) Representative *B2M* transcript abundance relative to *GAPDH* in HUVEC after 24 h treatment with indicated conditioned medium (left) and summary of n=8 treatments with conditioned medium from independent NK-cell stimulations (right). Statistical analyses performed with one-tailed Wilcoxon matched-pairs test. NS not significant, *p < 0.05, **p < 0.01.

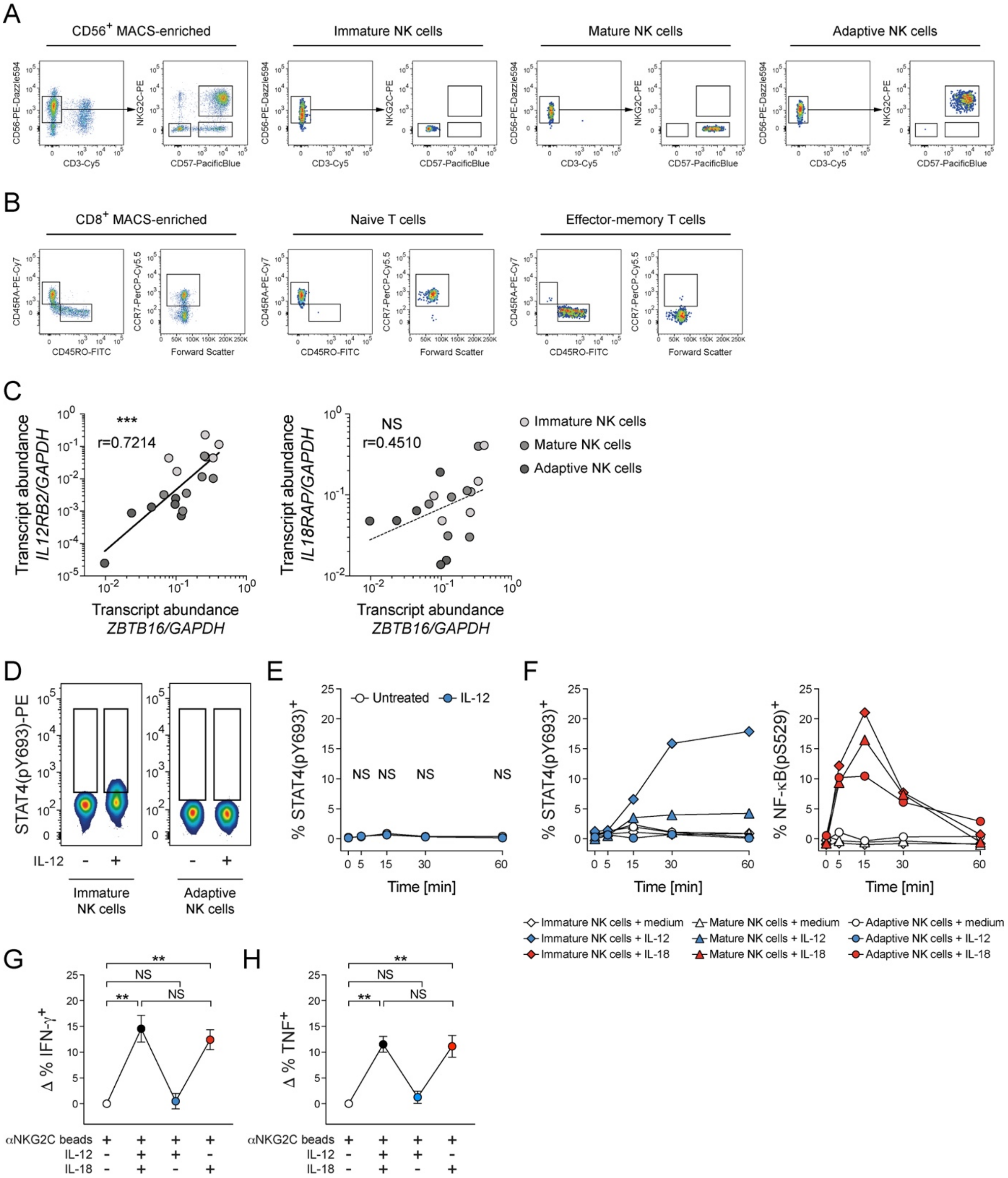

Supplementary Figure 3. Adaptive NK cells maintain *IL18RAP* expression and residual responsiveness to IL-18 (A-B) NK and T cells were sorted ex vivo from HCMV-seropositive healthy donors. **(A)** Representative sorting strategy to purify CD56^{+} MACS-enriched cells (left) into the indicated NK-cell sub-populations based on the expression of CD56, CD57, and NKG2C (right). **(B)** Representative sorting strategy to purify CD8^{+} MACS-enriched cells (left) into the indicated T-cell sub-populations based on the expression of CD45RA, CD45RO, and CCR7 (left). **(C)** Correlation of *IL12RB2* (left) and *IL18RAP* (right) transcript levels with *ZBTB16* transcript levels. Symbols indicate individual donors (n=6). Statistical analysis performed with Spearman correlation. **(D-F)** Immature, mature, and adaptive NK cells were FACS-purified to assess phosphorylation of

signaling molecules. **(D)** Representative staining of STAT4(pY693) in immature (left) and adaptive NKG2C^{+} (right) NK cells after treatment with medium or IL-12 for 15 min. **(E)** Summary of frequencies of STAT4(pY693)$^{+}$ adaptive NKG2C^{+} NK cells either treated with medium or IL-12 over time; n=4 individual donors. Symbols indicate mean and error bars SEM. Statistical analysis performed with repeated-measures two-way ANOVA with Bonferroni correction. **(F)** Frequencies of STAT4(pY693)$^{+}$ (left) and NF-κB(pS529)$^{+}$ (right) cells within the indicated NK-cell populations after treatment with IL-12 or IL-18, respectively; n=1 donor. **(G-H)** NK cells were cultured with beads coated with agonistic anti-NKG2C antibody in the presence of IL-12+18, IL-12, or IL-18. **(G)** Frequencies of ΔIFN-γ^{+} (calculated by subtraction of the frequency of IFN-γ^{+} cells in the presence of K562/HLA-E alone) and **(H)** ΔTNF^{+} adaptive NK cells; n=8 donors. Symbols indicate mean and error bars SEM. Statistical analysis performed with Friedman and Dunn's multiple comparison test. NS not significant, $^{**}p < 0.01$, $^{***}p < 0.001$.

1.2 Supplementary Tables

Antigen	Clone	Conjugate	Supplier	Cat#	RRID
N.A. (*isotype control*)	MOPC-21	Purified	BioLegend	400124	-
CCR7	G043H7	PerCP-Cy5.5	BioLegend	353219	AB_10915275
CD14	M5E2	BV510	BioLegend	301842	AB_2561946
CD19	HIB19	BV510	BioLegend	302242	AB_2561668
CD2	RPA-2.10	PerCP-Cy5.5	BioLegend	300216	AB_2561923
CD3	UCHT1	PE-Cy5	BioLegend	300410	AB_314064
CD3	UCHT1	Cy5	*In house*	-	-
CD3	UCHT1	V500	BD Biosciences	561416	AB_10612021
CD4	TT1	Alexa405	*In house*	-	-
CD45RA	HI100	PE-Cy7	BioLegend	304126	AB_10708879
CD45RO	UCHL1	FITC	*In house*	-	-
CD56	HCD56	PE-Dazzle594	BioLegend	318348	AB_2563564
CD57	HCD57	Pacific Blue	BioLegend	322316	AB_2063197
CD57	TBO1	Purified	ThermoFisher	14-0577-82	AB_2572855
CD8	GN11/134D7	Cy5	*In house*	-	-
E-selectin	HAE-1f	APC	BioLegend	336011	AB_2254490
HLA class I	W6/32	Pacific Blue	BioLegend	311418	AB_493669
ICAM-1	HA58	FITC	BioLegend	353107	AB_10898317
IFN-γ	45-15	PE-Vio770	Miltenyi	130-109-235	AB_2652240
IFN-γ	B27	Alexa700	BioLegend	506516	AB_961351
IFN-γ	B27	Purified	BioLegend	506513	AB_315446
ILT2	HP-F1	APC	ThermoFisher	17-5129-42	AB_1311214
Mouse IgM	RMM-1	BV605	BioLegend	406523	AB_2563358
NF-κB (pS529)	K10-895.12.50	PE	BD Biosciences	558423	AB_647222
NKG2A	REA110	Biotin	Miltenyi	130-098-819	AB_2655390
NKG2A	REA110	PE-Vio770	Miltenyi	130-105-647	AB_2655388
NKG2C	134591	Purified (biotinylated *in house*)	R&D Systems	MAB138-100	AB_2132982 (purified)
NKG2C	REA205	PE	Miltenyi	130-103-635	AB_2655394
NKG2C	REA205	VioBright-FITC	Miltenyi	130-104-731	AB_2655392
NKp30	AF29-4D12	eFluor450	ThermoFisher	48-3379-42	AB_2574058
Siglec-7	REA214	APC-Vio770	Miltenyi	130-101-009	AB_2657541
STAT4 (pY693)	38/p-Stat4	PE	BD Biosciences	558249	AB_397066
Streptavidin	-	BV785	BioLegend	405249	-
TNF	Mab11	BV605	BioLegend	502936	AB_2563884
TNF	Mab1	Purified	BioLegend	502804	AB_315252
VCAM-1	STA	PE	BioLegend	305805	AB_314561

Supplementary Table 1. Antibodies used for flow cytometry, coating of anti-biotin beads, and blocking experiments.

**Gene**	**Assay ID**	**Supplier**
GAPDH	Hs02786624_g1	ThermoFisher
IL12RB2	Hs00155486_m1	ThermoFisher
IL18RAP	Hs00187256_m1	ThermoFisher
ZBTB16	Hs00957433_m1	ThermoFisher
B2M	Hs00187842_m1	ThermoFisher
PSMB9	Hs00160610_m1	ThermoFisher
PKR/EIF2AK2	Hs00169345_m1	ThermoFisher

Supplementary Table 2. TaqMan gene expression assays used for quantitative RT-PCR.
